# To elucidate the mechanism of “Scrophulariae Radix–Fritillaria” in goiter by integrated metabolomics and serum pharmaco-chemistry

**DOI:** 10.3389/fphar.2024.1206718

**Published:** 2024-05-17

**Authors:** Lixin Chen, Wei Liang, Kun Zhang, Zishuo Wang, Wei Cheng, Wenlan Li

**Affiliations:** School of Pharmaceutical Sciences, Harbin University of Commerce, Harbin, Heilongjiang, China

**Keywords:** “Scrophulariae Radix–Fritillaria” drug pair, goiter, metabolomics, serum pharmacochemistry, UPLC-Q-TOF/MS

## Abstract

The pharmacodynamic substances in “Scrophulariae Radix–Fritillaria” and the molecular mechanisms underlying its therapeutic effects against goiter were analyzed through metabolomics and serum pharmaco-chemistry. A rat model of goiter was established using propylthiouracil (PTU), and the animals were treated using “Scrophulariae Radix–Fritillaria.” The efficacy of the drug pair was evaluated in terms of thyroid gland histopathology and blood biochemical indices. Serum and urine samples of the rats were analyzed by UPLC-Q-TOF/MS. Principal component analysis (PCA) and orthogonal partial least squares discriminant analysis (OPLS-DA) were performed to screen potential biomarkers in urine and the corresponding metabolic pathways. The blood components of “Scrophulariae Radix–Fritillaria” were also identified, and their correlation with urine biomarkers was analyzed in order to screen for potential bioactive compounds. “Scrophulariae Radix–Fritillaria” mitigated injury to thyroid tissues and normalized the levels of the thyroid hormones FT3, FT4, and TSH. We also identified 22 urine biomarkers related to goiter, of which 19 were regulated by “Scrophulariae Radix–Fritillaria.” Moreover, urine biomarkers are involved in tryptophan metabolism, steroid hormone biosynthesis, and beta-alanine metabolism, and these pathways may be targeted by the drug pair. In addition, 47 compounds of “Scrophulariae Radix–Fritillaria” were detected by serum pharmacochemistry, of which nine components, namely, syringic acid, paeonol, cedrol, and cis-ferulic acid, fetisinine, aucubigenin, linolenic acid, ussuriedine, and 5-(methylsulfanyl)pentanenitrile, were identified as potential effective substances against goiter. To summarize, we characterized the chemical components and mechanisms of “Scrophulariae Radix–Fritillaria” involved in the treatment of goiter, and our findings provide an experimental basis for its clinical application.

## 1 Introduction

Goiter is a common endocrine disease that currently affects nearly 10% of the global population (Carlé et al., 2014) and has a high cancerization rate (Saylam et al., 2013). It is characterized by the formation of solitary or multiple nodules in the thyroid, along with hyperplastic tissues. Goiter may be caused by excessive secretion of the thyroid-stimulating hormone, which repeatedly stimulates the glands to form proliferative nodules (Ziros et al., 2020) and is the result of insufficient iodine intake, autoimmunity, genetic and/or environmental disturbances, and mental stress (Kim et al., 2017; Führer, 2018; Vita et al., 2021). Most goiter patients have no obvious symptoms, while a few patients exhibit compression symptoms such as anterior neck discomfort, dyspnea, and dysphagia (Brinch et al., 2019). It currently can be treated using thyroid hormone inhibitors and surgery in severe cases. Although these therapeutic approaches can effectively relieve the symptoms of goiter, long-term medication or surgical resection can disrupt thyroid function, thus affecting the physical and mental wellbeing of patients.

Traditional Chinese medicine (TCM) is increasingly being considered for treatment of goiter since it has the advantages of consistent efficacy and few side effects. TCM classifies goiter as a “gall tumor” (Sheng et al., 2022) characterized by Qi stagnation, phlegm coagulation, blood stasis, and formation of a mass over time and considers emotional injury (Caye et al., 2020), diet, environment, and other factors as possible causes. The objectives of TCM in the treatment of gall tumor are Qi regulation, removal of phlegm, heat clearance, and detoxification. Scrophulariae Radix is derived from the dried root of *Scrophularia ningpoensis* Hemsl. and is documented in Chinese Pharmacopoeia as having a bitter, salty, and cold taste, along with heat-clearing and yin-nourishing effects. Fritillaria is a medicinal herb with a bitter and cold taste and has heat-clearing, detoxifying, and phlegm-dispersing effects. The combination of both herbs is effective against liver and kidney diseases that are characterized by yin deficiency, phlegm, and blood stasis and, therefore, can be used for treatment of gall tumors.

Metabolomics is the study of the changes found in metabolites in cells, tissues, and biological fluids and has been widely used to elucidate the composition and mechanisms of TCM formulations in various diseases (Zhang et al., 2015; Sun et al., 2021). Likewise, serum pharmacochemistry has also been applied to analyze and identify the drug components of TCM that are absorbed into the blood and determine the therapeutic efficacy and mechanistic basis (Ma et al., 2017). In this study, we combined metabolomics and serum pharmacochemistry to evaluate the therapeutic effects of the drug pair “Scrophulariae Radix–Fritillaria” in a rat model of goiter and identified the active compounds and metabolic pathways. Our findings provide an experimental basis for clinical application of “Scrophulariae Radix–Fritillaria” against goiter.

## 2 Materials and methods

### 2.1 Materials and reagents

Scrophularia Radix and Fritillaria were purchased from the Daowai district of Harbin Province Traditional Chinese Medicine company (Harbin, China) and certified by the School of Pharmacy of Harbin University of Commerce as conforming to the provisions of the Chinese Pharmacopoeia 2020 edition.

Propylthiouracil (PTU) was purchased from Beijing Century Aoke Biotechnology Co. Ltd. (Beijing, China). Levothyroxine sodium (LT-4) was purchased from Shenzhen Zhonglian Pharmaceutical Co. Ltd. (Shenzhen, China). FT3, FT4, and TSH ELISA kits were purchased from Jiangsu Baolai Biotechnology Co. Ltd. (Jiangsu, China). HPLC-grade acetonitrile and formic acid were purchased from Tedia Co. (United States of America).

### 2.2 Drug preparation for administration

Scrophulariae Radix and Fritillaria (at a 1:1 ratio) were ground to a powder and soaked in ten volumes of water for 30 min. The mixture was then boiled for 30 min and filtered. The filtered residue was reconstituted in ten volumes of water, decocted for 30 min, and then filtered. Both filtrates were combined, and water was added to adjust the relative concentration to 0.4 kg/L for testing in rats (Wei, 2010).

### 2.3 Establishment of the rat model of goiter and treatment regimen

A total of 40 SPF Wistar male rats weighing 160–180 g were purchased from Changchun Yisi Laboratory Animal Technology Co. Ltd. (Changchun, China). The animals were maintained at the Pharmacological Laboratory of Harbin University of Commerce (22°C ± 4°C, 50%–60% relative humidity, 12-h light/dark cycle). All experimental procedures were approved by the Experimental Animal Ethics Committee of Harbin University of Commerce (HSDYXY-20220017). After 1 week of adaptive feeding, the rats were randomly divided into the control, goiter model, TCM drug pair, and L-T4 groups (n = 10 each). To induce goiter, the animals were injected daily with PTU (20 mg kg^-1^·d^-1^) for 8 weeks (Liu et al., 2019); the rats in the control group received saline (1 mL/100 g) for the same duration. After successful induction of goiter, the animals were injected daily with 0.4 kg/L “Scrophulariae Radix–Fritillaria” or 7.8 μg kg^-1^·d^-1^ L-T4 along with PTU for 4 weeks (Wei, 2010).

### 2.4 Biological sample collection and preparation

After 4 weeks of drug intervention, the rats were placed in metabolic cages, and urine samples were collected over a 12-h period. The samples were centrifuged at 13,000 rpm for 15 min at 4°C, and the supernatant was collected and stored at −80°C. After thawing at room temperature, 100 μL of each sample was mixed as the quality control (QC). Thereafter, 100 μL of each sample and the QC was diluted using 200 μL of distilled water. After vortexing for 10 s, the samples were centrifuged at 13,000 rpm for 15 min at 4°C, and the supernatant was filtered through a 0.22-μm filter membrane for metabolomics analysis. Blood was drawn from the abdominal aorta of each rat after anesthetizing the animals. After resting for 30 min, the blood samples were centrifuged at 3,500 rpm for 15 min at 4°C, and the serum was stored at −80°C. One part was used for detecting biochemical indices, and the other part was used for serum pharmacochemical analysis. The samples were thawed at room temperature, and 1 mL of serum was diluted using 4 mL of methanol and vortexed for 60 s. The solution was centrifuged at 3,500 rpm for 15 min at 4°C, and the supernatant was dried with nitrogen and redissolved with 200 μL methanol. Finally, the samples were centrifuged at 3,500 rpm for 15 min at 4°C, and the supernatant was extracted for UPLC-Q-TOF/MS analysis. The thyroid tissues were collected after drawing blood, weighed, and fixed in 4% paraformaldehyde for histopathological examination.

### 2.5 Metabolomics study

#### 2.5.1 Chromatographic condition

Chromatography column: ACQUITY UPLCTM HSS C18 (100 mm × 2.1 mm, 1.8 μm) (Waters, United States of America). Column temperature: 40°C. Injection volume: 2 µL. Mobile phase A: 0.1% formic acid in acetonitrile and mobile phase B: 0.1% formic acid in water. Elution gradient conditions: 0–3 min, 1%–10% A; 3–5 min, 10%–20% A; 5–8.5 min, 20%–40% A; 8.5–9.5 min, 40%–99% A; 9.5–11.5 min, 99% A; and 11.5–12 min, 99%–1% A.

#### 2.5.2 Mass spectrometry conditions

Electrospray ion source (ESI) positive- and negative-ion modes were used for scanning. Capillary voltage: 3000 V (ESI^+^) and 2500 V (ESI^−^). Cone voltage: 30 V. Source temperature: 110°C. Extraction of cone voltage: 5.0 V. Desolvation gas temperature: 350°C. Cone gas flow: 50 L/h. Desolvation gas flow: 800 L/h. Leucine–enkephalin ([M + H]^+^ = 556.2771 and [M − H]^-^ = 554.2615) was used as a lock mass solution for accurate mass determination, and the mass scan range was 50–1,200 m/z.

#### 2.5.3 Data processing and analysis

The UPLC-Q-TOF/MS data were imported into Progenesis QI software for peak identification, peak alignment, and normalization and then imported into EZinfo 3.0 software for principal component analysis (PCA) and orthogonal partial least squares discriminant analysis (OPLS-DA) to screen for differential metabolites. The potential biomarkers were screened on the basis of variable importance in projection (VIP) score >1 and *p* < 0.05 and identified by HMDB database search. The MetaboAnalyst platform was used to analyze the metabolic pathways most relevant to goiter.

### 2.6 Chemical characterization of “Scrophulariae Radix–Fritillaria”

#### 2.6.1 Sample preparation

Briefly, 2 g each of Scrophulariae Radix and Fritillaria was extracted twice for 70 min with 40 mL of 70% ethanol, and the extracts were combined. After concentrating to a thick paste, the extracts were dried to powder form in a 70°C water bath. The powdered drug was placed in a volumetric flask, sonicated for 30 min with methanol, and then dissolved in methanol. The solution was centrifuged at 13,000 rpm for 15 min at 4°C, and the supernatant was filtered through a 0.22-µm filter membrane for UPLC-Q-TOF/MS analysis.

#### 2.6.2 Chromatographic condition

Chromatography column: ACQUITY UPLCTM HSS C18 (100 mm × 2.1 mm, 1.8 μm) (Waters, United States of America). Column temperature: 5°C. Injection volume: 2 µL. Mobile phase A: 0.1% formic acid in acetonitrile and mobile phase B: 0.1% formic acid in water. Elution gradient conditions: 0–29 min, 1%–99% A; 29–29.5 min, 99% A; and 29.5–30 min, 99%–1% A.

#### 2.6.3 Mass spectrometry conditions

Electrospray ion source (ESI) positive- and negative-ion modes were used for scanning. Capillary voltage: 3000 V (ESI^+^) and 2500 V (ESI^−)^. Cone voltage: 30 V. Source temperature: 110°C. Extraction of cone voltage: 5.0 V. Desolvation gas temperature: 350°C. Cone gas flow: 50 L/h. Desolvation gas flow: 800 L/h. Leucine–enkephalin ([M + H]^+^ = 556.2771 and [M − H]^-^ = 554.2615) was used as a lock mass solution for accurate mass determination. Mass scan range: 50–1,200 m/z.

#### 2.6.4 Data processing and analysis

The serum data of the model and drug-treated groups obtained by UPLC-Q-TOF/MS were imported into Progenesis QI software, and the metabolites were identified by matching the retention time and mass spectrometry characteristics.

### 2.7 Correlation analysis

The correlation analysis method of biomarkers and components was established, and the component and biomarker data were imported into the OmicStudio data platform for Pearson’s correlation analysis. The potential biomarkers were screened on the basis of correlation coefficient 0.7≤|r|≤1 and *p* < 0.05.

### 2.8 Statistical analysis

Statistical analysis was performed using SPSS 20.0 software. The data are expressed as ( $X\;\bar{\pm }S$ ). Single-factor analysis of variance (ANOVA) was used to compare the groups, and *t*-test was used for intra-group comparison. *p* < 0.05 was considered statistically significant.

## 3 Results

### 3.1 “Scrophulariae Radix–Fritillaria” mitigated the symptoms of goiter in a rat model

**FIGURE 1:**
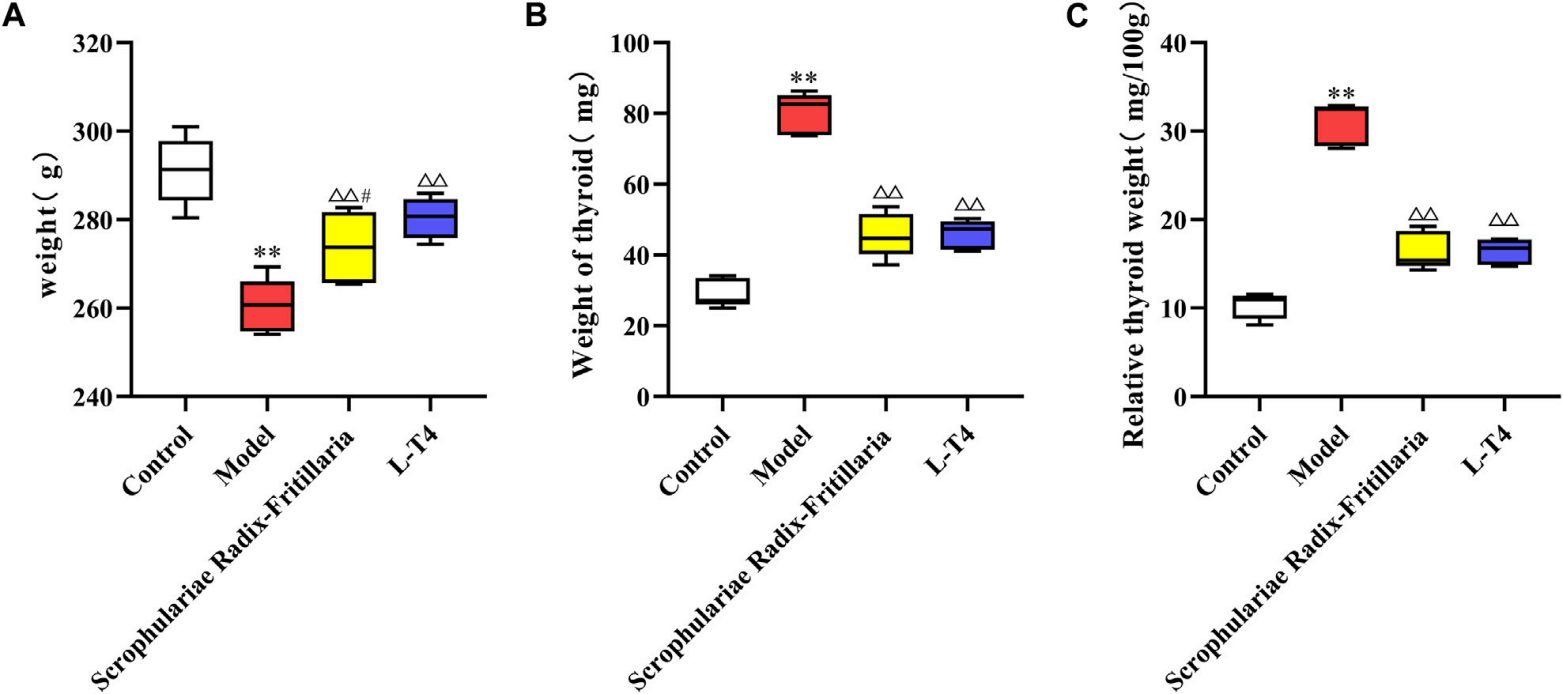
Comparison of weight and thyroid weight in each group. Note: compared with the control group, *p < 0.05 and **p < 0.01; compared with the model group, △*p* < 0.05 and △△*p* < 0.01; compared with the L-T4 group, #*p* < 0.05 and ##*p* < 0.01. **(A)**: Weight (g) **(B)**: Weight of thyroid (mg) **(C)**: Relative thyroid weight (mg/100mg).

Continuous exposure to PTU significantly increased the body weight, thyroid weight, and relative thyroid weight of rats compared to that of the control group animals (*p* < 0.01), which was indicative of successful induction of the goiter model. However, treatment with “Scrophulariae Radix–Fritillaria” and L-T4 reduced the above indices to near normal levels (*p* < 0.01 compared to the model group), suggesting that both drugs can alleviate the symptoms of goiter (Figure 1).

### 3.2 Histopathological results of the rat thyroid gland

**FIGURE 2:**
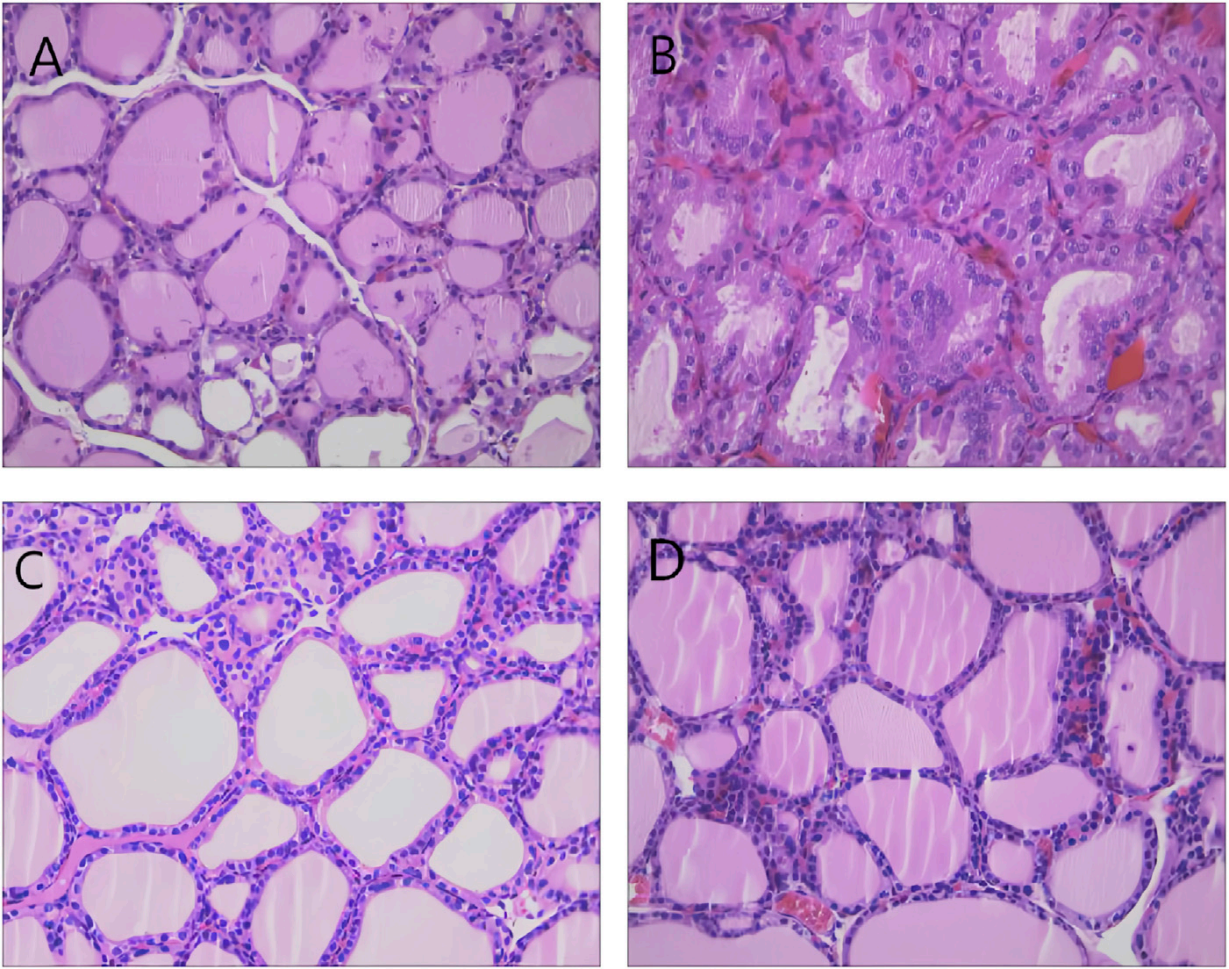
Representative images showing thyroid histopathology in each group (HE staining × 400). Note: **(A)** control group; **(B)** model group; **(C)** L-T4 group; and **(D)** “Scrophulariae Radix–Fritillaria” drug pair group.

Histological examination of thyroid tissues from the control group indicated a single layer of cuboid or short columnar thyroid follicular epithelial cells around the thyroid follicles, along with uniformly distributed glial cells. In the model group, the thyroid follicular epithelial cells were hypertrophic and formed a columnar stratified structure, and the follicular cavity was diminished. The glial cells in the follicular cavity were either reduced or altogether absent, and new follicular cavities were formed, further confirming the successful establishment of the model. The thyroid follicles recovered to medium size, the follicular cavity expanded, and the amount of colloid in the cavity increased after administering different drugs, indicating effective drug intervention (Figure 2).

### 3.3 Results of serum biochemical indexes

**FIGURE 3:**
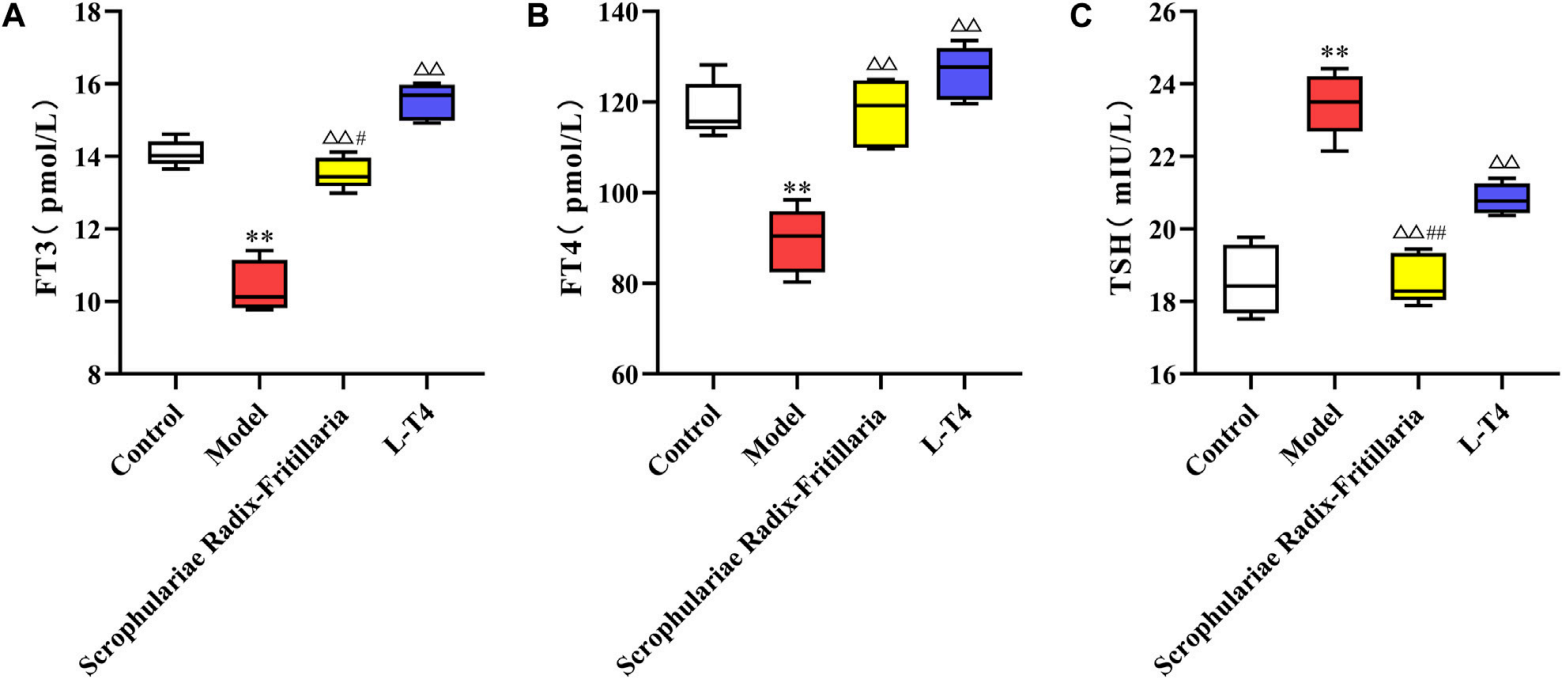
Changes in FT3, FT4, and TSH contents in each group of rats. Note: compared with the control group, *p < 0.05 and **p < 0.01; compared with the model group, △*p* < 0.05 and △*p* < 0.01; and compared with the L-T4 group, #*p* < 0.05 and ##*p* < 0.01. **(A)**: FT3 (pmol/L) **(B)**: FT4 (pmol/L) **(C)**: TSH (mIU/L).

Consistent with the above results, serum FT3 and FT4 levels were decreased, while TSH was elevated in the model group (*p* < 0.01). However, “Scrophulariae Radix–Fritillaria” and L-T4 normalized the levels of thyroid hormones, and the difference was significant compared to that in the model group (*p* < 0.01 for FT3 and FT4, *p* < 0.05 for TSH). Furthermore, the therapeutic effects of both drugs were similar (Figure 3).

### 3.4 Results of metabonomics analysis

#### 3.4.1 Metabolic profiling analysis

**FIGURE 4:**
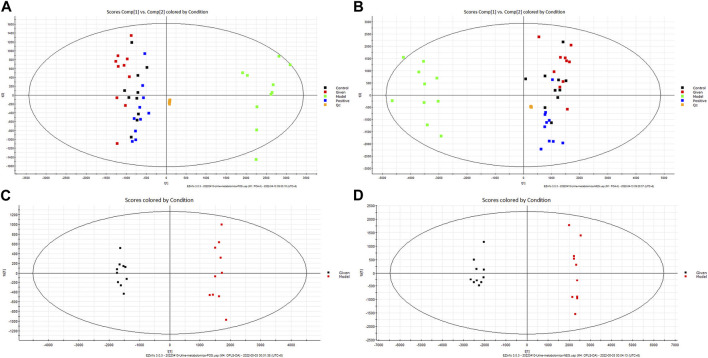
Multivariate tests. Note: **(A)** PCA score chart (positive-ion mode). **(B)** PCA score chart (negative-ion mode). **(C)** OPLS-DA score chart (positive-ion mode). **(D)** OPLS-DA score chart (negative-ion mode).

The urine metabolites in different groups were identified by UPLC-Q-TOF/MS in the positive- and negative-ion modes. Unsupervised PCA of the QC sample data showed good aggregation and stability. Furthermore, the metabolic profiles of different groups showed significant clustering. The control group and model group formed clearly distinct clusters, which suggested that goiter led to significant changes in small endogenous metabolites and that our model was reliable. In contrast, the metabolic trajectory of samples from the “Scrophulariae Radix–Fritillaria” group was close to that from the control group, indicating that the drug pair can effectively improve the metabolic disruption caused due to goiter (Figures 4A, B).

To further screen for potential biomarkers, the model group was further analyzed by supervised OPLS-DA. According to the OPLS-DA score map, the metabolic profiles of the goiter model and control groups were completely separated (Figures 4C, D). Furthermore, R2Y = 0.979 and Q2 = 0.971 in the positive-ion mode and R2Y = 0.972 and Q2 = 0.959 in the negative-ion mode indicated that the model was stable and reliable without overfitting.

#### 3.4.2 Biomarker identification

**FIGURE 5:**
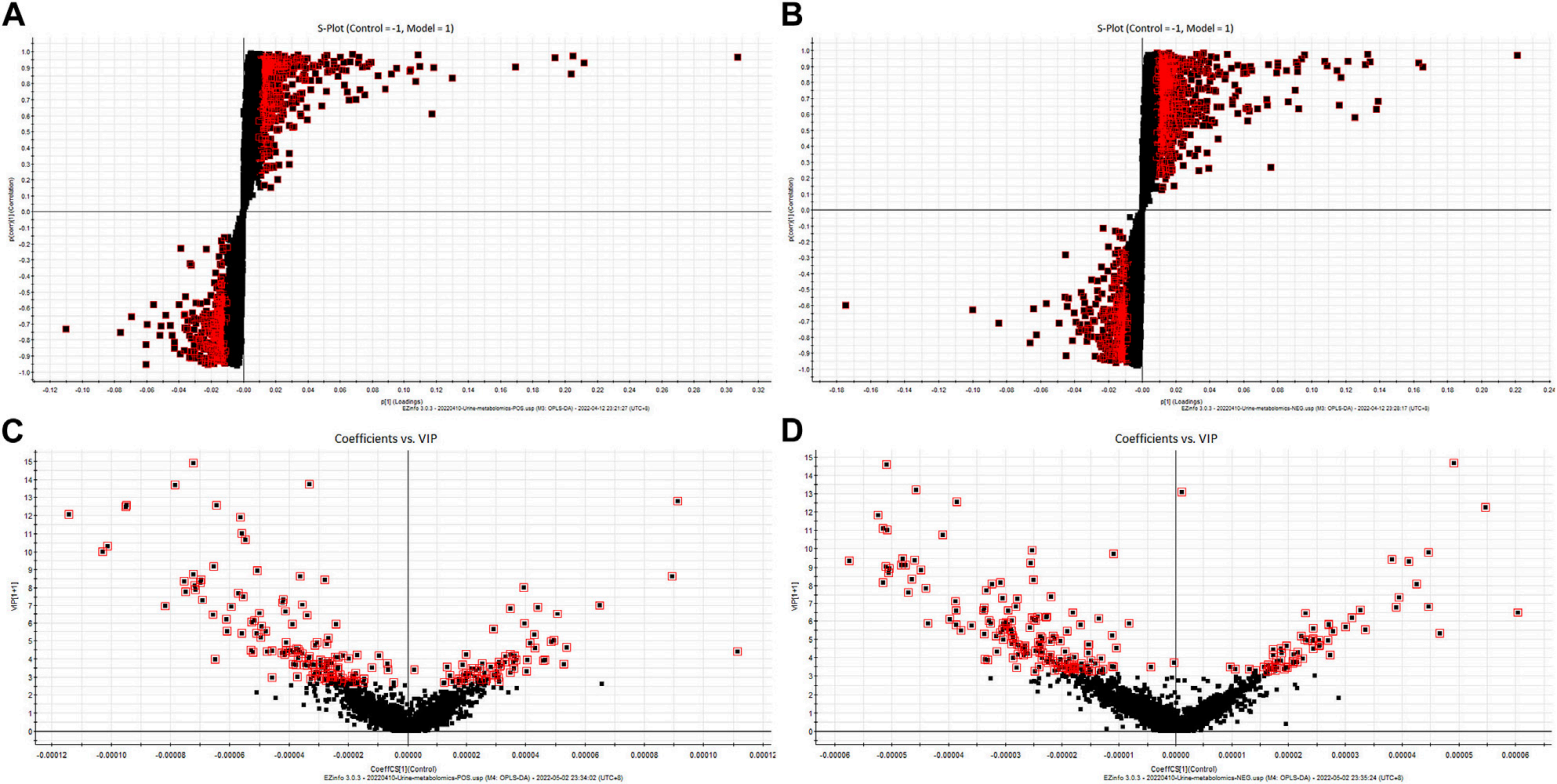
S-plot and VIP-plot of the rat urine metabolic profile. **(A)**: PCA score chart (positive-ion mode) **(B)**: PCA score chart (negative-ion mode) **(C)**: OPLS-DA score chart (positive-ion mode) **(D)**: OPLS-DA score chart (negative-ion mode).

The metabolites that significantly contributed to the distinct metabolomics profiles of the control and model groups were screened using the S-plot and VIP-plot, with VIP >1 and *p* < 0.05 as the thresholds. We identified 22 biomarkers in the goiter model by matching the mass spectrometry information with the HMDB database. Compared to the control group, seven metabolites (ribothymidine, serotonin, beta-alanine, quinoline-4,8-diol, 4-(2-aminophenyl)-2,4-dioxobutanoic acid, pimelic acid, and ascorbic acid) showed increased levels and 15 metabolites (isoleucylvaline, 5-methylcytosine, dihydrobiopterin, succinyladenosine, tyrosyl-proline, xanthurenic acid, androstenedione, cortisol, uric acid, N2-succinyl-L-ornithine, inosine, N-acetylornithine, 4-oxo-1-(3-pyridyl)-1-butanone, 2-methoxyestrone 3-glucuronide, and aldosterone) showed decreased levels in the goiter model group. Nineteen of the above biomarkers were restored to normal levels in the “Scrophulariae Radix–Fritillaria” group (Figure 5; Table 1).

**TABLE 1:** Information on potential biomarkers of urine metabolism in goiter rats.

No	Metabolite	RT	m/z	Formula	VIP	MS/MS	Trend	Scan mode
1	Ribothymidine	0.72	259.09	C^10^H^14^N^2^O^6^	2.54	259.09, 153.06, 127.05	↑	+
2	Isoleucylvaline	0.76	231.17	C^11^H^22^N^2^O^3^	3.30	231.17, 144.07, 129.10, 101.06	↓	+
3	5-Methylcytosine	0.97	126.07	C^5^H^7^N^3^O	1.23	126.07, 109.04, 81.04	↓	+
4	Dihydrobiopterin	1.26	240.11	C^9^H^13^N^5^O^3^	1.66	240.11, 222.10, 195.08, 165.07, 164.06	↓	+
5	Serotonin	1.91	177.10	C^10^H^12^N^2^O	1.38	177.10, 159.09	↑	+
6	Beta-Alanine	2.55	90.06	C^3^H^7^NO^2^	1.16	90.06, 73.03, 70.03	↑	+
7	Succinyladenosine	2.62	384.12	C^14^H^1^N^5^O^8^	2.79	384.12, 252.07, 234.06, 216.05, 162.08, 121.05	↓	+
8	Tyrosyl-Proline	2.72	279.13	C^14^H^1^N^2^O^4^	1.78	279.13, 164.07, 146.06, 91.05	↓	+
9	Xanthurenic acid	3.14	206.05	C^10^H^7^NO^4^	3.57	206.05, 188.03, 178.05, 160.04, 132.04	↓	+
10	Quinoline-4,8-diol	3.88	162.06	C^9^H^7^NO^2^	3.74	162.06, 144.04, 134.06, 116.05	↑	+
11	Androstenedione	5.09	287.20	C^19^H^26^O^2^	1.49	287.20, 269.19, 241.16, 119.09, 91.05	↓	+
12	Cortisol	7.13	363.21	C^21^H^30^O^5^	1.15	363.22, 235.13, 217.12, 177.09	↓	+
13	Uric acid	0.89	167.02	C^5^H^4^N^4^O^3^	7.67	167.02, 166.01, 96.02	↓	-
14	N2-Succinyl-L-ornithine	0.93	231.10	C^9^H^16^N^2^O^5^	1.81	231.10, 187.11, 173.09, 171.08	↓	-
15	Inosine	1.60	267.07	C^10^H^12^N^4^O^5^	4.26	267.07, 148.04, 135.03	↓	-
16	N-Acetylornithine	1.66	173.09	C^7^H^14^N^2^O^3^	1.78	173.09, 155.08, 131.08, 129.10, 126.06, 112.08	↓	-
17	4-(2-Aminophenyl)-2,4-dioxobutanoic acid	3.95	206.05	C^10^H^9^NO^4^	1.43	206.05, 191.04, 188.04, 162.06, 160.04, 132.05	↑	-
18	Pimelic acid	4.20	159.07	C^7^H^12^O^4^	3.15	159.07, 115.08, 113.06, 97.07, 95.05	↑	-
19	4-Oxo-1-(3-pyridyl)-1-butanone	4.73	162.06	C^9^H^9^NO^2^	5.14	162.06, 134.06, 120.05, 95.01, 85.03	↓	-
20	Ascorbic acid	4.73	175.02	C^6^H^8^O^6^	3.95	175.03, 161.05, 157.01, 129.02, 117.02, 115.00, 113.02, 103.00, 99.01, 85.03, 71.01	↑	-
21	2-Methoxyestrone 3-glucuronide	7.48	447.20	C^24^H^32^O^8^	1.47	447.20, 385.17, 269.14, 247.13, 205.09, 149.06	↓	-
22	Aldosterone	7.79	359.19	C^21^H^28^O^5^	1.10	359.19, 331.19, 291.16, 205.12	↓	-

#### 3.4.3 Metabolic pathway analysis

**FIGURE 6:**
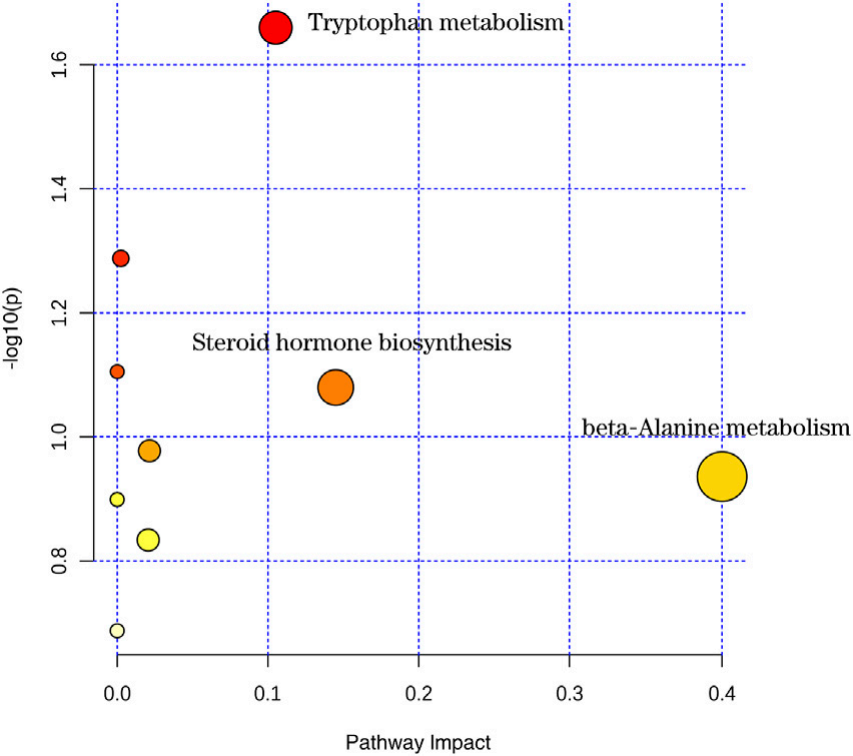
Metabolic pathway map.

The 22 potential biomarkers identified in the urine of the goiter model were imported into MetaboAnalyst software, and nine metabolic pathways were identified, i.e., those related to tryptophan metabolism, steroid hormone biosynthesis, arginine biosynthesis, purine metabolism, pantothenate and CoA biosynthesis, beta-alanine metabolism, propanoate metabolism, folate biosynthesis, and pyrimidine metabolism. With impact >0.1 as the threshold, tryptophan metabolism, steroid hormone biosynthesis, and beta-alanine metabolism were the three most affected metabolic pathways in goiter and were restored by “Scrophulariae Radix–Fritillaria” (Figure 6). To summarize, several endogenous metabolites are disrupted during the progression of goiter, and the corresponding metabolic pathways are likely involved in its pathogenesis and targeted by the drug pair.

### 3.5 Analysis of the serum components of “Scrophulariae Radix–Fritillaria”

#### 3.5.1 Component analysis of “Scrophulariae Radix–Fritillaria” drug *in vitro*

**FIGURE 7:**
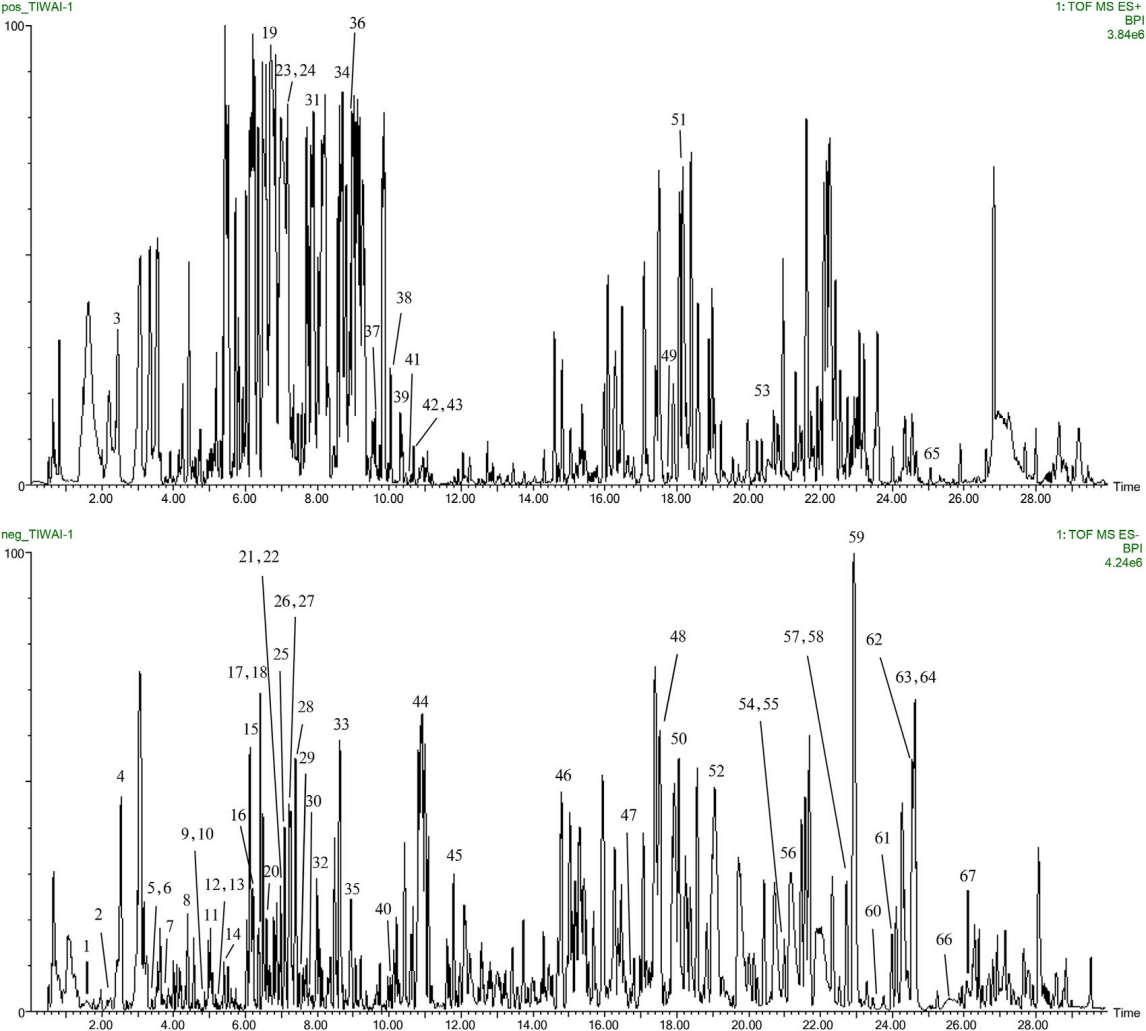
Chromatographic diagram of BPI of the “Scrophulariae Radix–Fritillaria” drug pair.

The UPLC-Q-TOF/MS technique was used to detect and analyze the extraction solution of the drug pair “Scrophulariae Radix–Fritillaria”, and the ion flow diagrams of “Scrophulariae Radix–Fritillaria” drug pair samples in positive- and negative-ion modes were obtained (Figure 7). According to the mass-to-charge ratio, molecular ion peak, and secondary mass spectrometry cleavage of the compounds and comparison with the data in the literature, 67 components of the “Scrophulariae Radix–Fritillaria” drug pair were identified. There were 18 alkaloids, 12 terpenoids, 9 phenylpropanes, 9 organic acids, 2 flavonoids, 5 volatile compounds, 3 acetophenones, 3 sugars, and 6 other compounds, of which 45 were derived from Scrophulariae Radix and 22 from Fritillaria (Figure 7; Table 2).

**TABLE 2:** Components of “Scrophulariae Radix–Fritillaria” drug pairs *in vitro*.

No	Component	Formula	Ion mode	Calculated mass	Measured mass	Error	Rt	Source
1	Vanillin	C_16_H_14_O_5_	M − H	152.15	151.04	−2.62	1.81	Scrophulariae Radix
2	Geniposide	C_16_H_30_O_2_	M − H	388.37	387.13	3.55	10.60	Scrophulariae Radix
3	Liquiritigenin	C_16_H_32_O_2_	M + H	256.25	257.08	0.06	10.65	Scrophulariae Radix
4	[(1S, 2R, 6R, 8R)-8-hydroxy-6-methyl- 2-[3,4,5-trihydroxy- 6-(hydroxymethyl)oxa n-2-yl]oxy-9,10- dioxatricyclo [4.3.1.0 3,8]decan-2-yl]methyl benzoate	C_15_H_24_O_9_	M + H	482.50	483.19	9.13	10.71	Scrophulariae Radix
5	(−)-Nissolin	C_9_H_18_O_2_	M − H	286.28	285.08	−1.87	14.89	Scrophulariae Radix
6	9-Hexadecenoic acid	C_10_H_12_O	M − H	254.41	253.22	1.10	16.72	Scrophulariae Radix
7	Palmitic acid	C_18_H_34_O_2_	M − H	256.42	255.23	1.66	17.85	Scrophulariae Radix
8	Ajugol	C_8_H_10_O_3_	M + H	348.34	349.15	0.60	17.89	Scrophulariae Radix
9	Nonanoic acid	C_16_H_14_O_5_	M − H	158.24	157.12	3.50	17.99	Scrophulariae Radix
10	Estragole	C_16_H_30_O_2_	M + H	148.20	149.10	7.76	18.26	Scrophulariae Radix
11	Elaidic acid	C_16_H_32_O_2_	M − H	282.50	281.25	1.89	18.90	Scrophulariae Radix
12	Hydroxytyrosol	C_15_H_24_O_9_	M − H	154.16	153.06	1.85	2.33	Scrophulariae Radix
13	3-Methoxyphenol	C_8_H_8_O_3_	M + H	124.14	125.06	8.85	2.40	Scrophulariae Radix
14	2-Methoxy-4-methylphenol	C_17_H_24_O_10_	M − H	138.16	137.06	0.43	2.54	Scrophulariae Radix
15	Cedrol	C_15_H_12_O_4_	M + H	222.37	223.21	2.76	20.48	Scrophulariae Radix
16	Linolenic acid	C_23_H_30_O_11_	M − H	278.40	277.22	7.32	21.73	Scrophulariae Radix
17	Myristic acid	C_7_H_8_O_2_	M − H	228.37	227.20	2.71	21.80	Scrophulariae Radix
18	Oleanonic acid	C_8_H_1_O_2_	M − H	454.70	453.34	2.55	22.96	Scrophulariae Radix
19	Linoleic acid	C_1_H_26_O	M − H	280.40	279.23	2.59	22.98	scrophulariae radix
20	5-Hydroxymethylfurfural	C_18_H_30_O_2_	M − H	126.11	125.02	2.47	3.21	Scrophulariae Radix
21	Aucubigenin	C_14_H_28_O_2_	M − H	184.19	183.07	0.54	3.25	Scrophulariae Radix
22	Vanillic acid	C_3_H_46_O_3_	M − H	168.15	167.04	8.28	3.92	Scrophulariae Radix
23	6-O-methyl catalpol	C_1_H_32_O_2_	M − H	376.36	375.13	0.49	4.52	Scrophulariae Radix
24	Methoxycinnamic acid	C_6_H_6_O_3_	M − H	178.18	177.06	0.91	4.89	Scrophulariae Radix
25	2′-Hydroxy-5′-methoxyacetophenone	C_9_H_12_O_4_	M − H	166.17	165.06	−2.24	4.90	Scrophulariae Radix
26	Acetophenone	C_8_H_8_O_4_	M − H	120.15	119.05	−0.75	4.95	Scrophulariae Radix
27	cis-Cinnamic acid	C_16_H_24_O_10_	M − H	148.16	147.05	2.21	5.28	Scrophulariae Radix
28	Paeonol	C_9_H_10_O_3_	M − H	166.17	165.06	10.39	5.28	Scrophulariae Radix
29	Aucubin	C_15_H_22_O_9_	M − H	346.33	345.12	2.62	5.48	Scrophulariae Radix
30	cis-Ferulic acid	C_10_H_10_O_4_	M − H	194.18	193.05	3.75	5.97	Scrophulariae Radix
31	Asparagine	C_4_H_8_N_2_O_3_	M − H	132.12	131.05	−0.56	6.21	Scrophulariae Radix
32	Angroside C	C_36_H_48_O_19_	M − H	784.80	783.27	−7.21	6.29	Scrophulariae Radix
33	Ningposide C	C_17_H_20_O_8_	M − H	352.30	351.11	3.68	6.68	Scrophulariae Radix
34	Octanoic acid	C_8_H_16_O_2_	M − H	144.21	143.11	2.62	6.89	Scrophulariae Radix
35	Ningpogenin	C_9_H_14_O_3_	M − H	170.21	169.09	2.23	6.89	Scrophulariae Radix
36	Homovanillyl alcohol	C_9_H_12_O_3_	M − H	168.19	167.07	−9.88	7.08	Scrophulariae Radix
37	Ningposide A	C_18_H_22_O_9_	M − H	382.40	381.12	1.34	7.15	Scrophulariae Radix
38	Ningposide B	C_18_H_22_O_9_	M − H	382.40	381.12	2.51	7.63	Scrophulariae Radix
39	Cistanoside D	C_31_H_40_O_15_	M − H	652.60	651.23	−0.59	8.06	Scrophulariae Radix
40	D-Fructose	C_6_H_12_O_6_	M − H	180.16	179.06	−0.34	8.60	Scrophulariae Radix
41	Scropolioside A	C_35_H_44_O_18_	M + H	752.70	753.26	0.35	8.93	Scrophulariae Radix
42	D-glucose	C_6_H_12_O_6_	M − H	180.16	179.07	2.35	6.26	Scrophulariae Radix
43	Syringic acid	C_9_H_10_O_5_	M − H	198.17	197.04	3.58	7.11	Scrophulariae Radix
44	Eugenol	C_10_H_12_O_2_	M − H	164.20	163.08	3.29	11.04	Scrophulariae Radix
45	Lupeol	C_30_H_50_O	M − H	426.70	425.36	3.38	25.74	Scrophulariae Radix
46	Puqietinedione	C_28_H_45_NO_2_	M + H	427.70	428.35	2.85	10.03	Fritillaria
47	Zhebeirine	C_27_H_43_NO_2_	M + H	413.60	414.34	1.88	10.30	Fritillaria
48	Solanidine	C_27_H_43_NO	M + H	397.60	398.34	3.96	10.67	Fritillaria
49	Fetisinine	C_27_H_39_NO_3_	M − H	425.60	424.28	−1.75	21.70	Fritillaria
50	Peimisine	C_27_H_41_NO_3_	M − H	427.60	426.30	2.18	23.03	fFritillaria
51	Ussuriedine	C_27_H_37_NO_3_	M − H	423.60	422.27	9.61	23.97	Fritillaria
52	Suchengbeisine	C_27_H_43_NO_3_	M − H	429.60	428.32	−1.49	24.47	Fritillaria
53	Peiminine	C_27_H_43_NO_3_	M − H	429.60	428.32	−1.58	24.57	Fritillaria
54	Kaur-15-en-17-ol	C_20_H_32_O	M + H	288.50	289.25	4.47	25.11	Fritillaria
55	Peimine	C_27_H_45_NO_3_	M − H	431.70	430.33	0.29	26.17	Fritillaria
56	Isoverticine	C_27_H_45_NO_3_	M + H	431.70	432.35	7.41	6.66	Fritillaria
57	Petilidine	C_27_H_45_NO_2_	M + H	415.70	416.35	5.17	6.94	Fritillaria
58	Puqiedine	C_27_H_45_NO_2_	M + H	415.70	416.35	5.17	6.94	Fritillaria
59	Pingbeimine C	C_27_H_43_NO_6_	M − H	477.60	476.30	−10.54	7.91	Fritillaria
60	Delavinone	C_27_H_43_NO_2_	M + H	413.60	414.34	4.85	7.94	Fritillaria
61	Impranine	C_28_H_45_NO_2_	M + H	427.70	428.35	3.55	8.61	Fritillaria
62	Spongipregnoloside A	C_33_H_52_O_11_	M − H	624.80	623.34	−4.57	8.80	Fritillaria
63	Ebeiedinone	C_27_H_43_NO_2_	M + H	413.60	414.34	2.96	9.61	Fritillaria
64	Adenosine	C_10_H_13_N_5_O_4_	M − H	267.24	266.10	1.20	7.51	Fritillaria
65	Puqiedinone	C_27_H_43_NO_2_	M − H	413.60	412.29	4.02	23.60	Fritillaria
66	Imperialine	C_27_H_43_NO_3_	M − H	429.60	428.32	3.72	24.52	Fritillaria
67	Smilaxchinoside C	C_45_H_72_O_18_	M − H	901.00	899.51	3.51	11.76	Fritillaria

#### 3.5.2 Analysis of serum components of “Scrophulariae Radix–Fritillaria”

To further identify the bioactive components of “Scrophulariae Radix–Fritillaria” involved in goiter intervention, we analyzed the serum samples of the model and treated groups and matched the primary and secondary mass spectrometry data with the chemical components of “Scrophulariae Radix–Fritillaria.” A total of 47 components of the drug pair were detected in the serum samples, of which 12 components were derived from Fritillaria and 35 from Scrophulariae Radix (Tables 3, 4).

**TABLE 3:** Prototype components.

Rt	Component	Formula	Ion mode	Calculated mass	Measured mass	Error	MS/MS	Source
5.03	2′-Hydroxy-5′-methoxyacetophenone	C_9_H_10_O_3_	[M − H]	166.17	165.06	3.74	147.05, 119.05	Scrophulariae Radix
2.40	3-Methoxyphenol	C_7_H_8_O_2_	[M − H]	124.14	123.05	−2.72	80.05, 77.04	Scrophulariae Radix
4.53	6-O-methyl catalpol	C_16_H_24_O_10_	[M + H]	376.36	377.15	1.80	375.13, 332.11, 312.12, 301.09	Scrophulariae Radix
16.70	9-Hexadecenoic acid	C_16_H_30_O_2_	[M − H]	254.41	253.22	2.39	253.22, 237.19, 179.14	Scrophulariae Radix
4.94	Acetophenone	C_8_H_8_O	[M − H]	166.17	119.05	−1.46	119.05, 101.02	Scrophulariae Radix
5.64	Angroside C	C_36_H_48_O_19_	[M − H]	784.80	783.28	10.70	783.27, 665.21, 649.24, 607.22	Scrophulariae Radix
20.46	Cedrol	C_15_H_26_O	[M + H]	222.37	223.21	6.70	203.18, 189.16, 149.13	Scrophulariae Radix
5.18	cis-Cinnamic acid	C_9_H_8_O_2_	[M − H]	148.16	147.05	0.90	147.05, 103.06	Scrophulariae Radix
3.94	cis-Ferulic acid	C_10_H_10_O_4_	[M − H]	194.18	193.05	1.52	193.05, 178.03, 175.04, 149.06, 147.04, 134.04	Scrophulariae Radix
18.97	Elaidic acid	C_18_H_34_O_2_	[M − H]	282.50	281.25	2.27	281.24, 224.07	Scrophulariae radix
22.55	Estragole	C_10_H_12_O	[M − H]	148.20	147.08	2.61	149.10, 109.07, 107.05	Scrophulariae adix
17.68	Fetisinine	C_27_H_39_NO_3_	[M − H]	425.60	424.28	−5.70	424.29, 389.27, 277.22, 277.22	Fritillaria
6.89	Isoverticine	C_27_H_45_NO_3_	[M + H]	431.70	432.35	0.81	398.31, 396.33	Fritillaria
26.63	Kaur-15-en-17-ol	C_20_H_32_O	[M + H]	288.50	289.25	2.25	271.24, 243.21	Fritillaria
22.96	Linoleic acid	C_18_H_32_O_2_	[M − H]	280.40	279.23	1.46	261.22, 183.01	Scrophulariae Radix
21.69	Linolenic acid	C_18_H_30_O_2_	[M + H]	278.40	279.23	5.77	259.21, 233.23, 205.20	Scrophulariae Radix
9.45	Liquiritigenin	C_15_H_12_O_4_	[M − H]	256.25	255.07	0.26	119.05, 89.37	Scrophulariae Radix
21.77	Myristic acid	C_14_H_28_O_2_	[M − H]	228.37	227.20	1.49	227.20, 179.14	Scrophulariae Radix
6.90	Ningpogenin	C_9_H_14_O_3_	[M − H]	170.21	169.09	1.63	169.08, 141.09, 137.06, 123.08	Scrophulariae Radix
23.08	Oleanonic acid	C_30_H_46_O_3_	[M − H]	454.70	453.34	0.40	407.33, 279.24	Scrophulariae Radix
5.28	Paeonol	C_9_H_10_O_3_	[M − H]	166.17	165.06	3.29	165.06, 114.02, 111.01	Scrophulariae Radix
17.90	Palmitic acid	C_16_H_32_O_2_	[M − H]	256.42	255.23	3.11	255.23, 196.04	Scrophulariae Radix
26.13	Peimine	C_27_H_45_NO_3_	[M − H]	431.70	430.34	5.47	430.33, 383.35	Fritillaria
23.00	Peimisine	C_27_H_41_NO_3_	[M − H]	427.60	426.30	−1.37	426.30, 392.32	Fritillaria
10.28	Pingbeimine C	C_27_H_43_NO_6_	[M − H]	477.60	476.30	−1.05	476.30, 171.10	Fritillaria
17.74	Ussuriedine	C_27_H_37_NO_3_	[M − H]	423.60	422.27	−5.86	403.25, 125.10	Fritillaria
17.55	Puqiedine	C_27_H_45_NO_2_	[M + H]	415.70	416.36	1.04	361.28, 343.20, 240.10	Fritillaria
28.46	Methoxycinnamic acid	C_10_H_10_O_3_	[M + H]	178.18	179.07	1.38	125.00, 86.10	Scrophulariae Radix
7.15	Aucubigenin	C_9_H_12_O_4_	[M + H]	184.19	185.08	−0.68	185.08, 171.06	Scrophulariae Radix
2.76	Vanillic acid	C_8_H_8_O_4_	[M − H]	168.15	167.03	−1.87	164.84, 160.84, 125.87	Scrophulariae Radix
7.51	Adenosine	C_10_H_13_N_5_O_4_	[M − H]	267.24	266.11	1.20	162.84, 160.84, 141.87	Fritillaria
23.60	Puqiedinone	C_27_H_43_NO_2_	[M − H]	413.60	412.29	4.01	303.23, 257.22	Fritillaria
24.52	Imperialine	C_27_H_43_NO_3_	[M − H]	429.60	428.32	3.72	428.32, 384.33, 330.25	Fritillaria
11.76	Smilaxchinoside C	C_45_H_72_O_18_	[M − H]	901.00	899.51	3.51	471.22, 405.27, 175.06	Fritillaria
6.27	D-Glucose	C_6_H_12_O_6_	[M − H]	180.16	179.07	2.35	162.84, 60.84	Scrophulariae Radix
7.11	Syringic acid	C_9_H_10_O_5_	[M − H]	198.17	197.04	3.58	143.87, 141.87	Scrophulariae Radix
11.05	Eugenol	C_10_H_12_O_2_	[M − H]	164.20	163.08	3.29	113.03, 85.03	Scrophulariae Radix
25.74	Lupeol	C_30_H_50_O	[M − H]	426.70	425.36	3.38	279.23, 255.23	Scrophulariae Radix

**TABLE 4:** Metabolic components.

Rt	Component	Formula	Ion mode	Calculated mass	Measured mass	Error	MS/MS	Source
11.43	Anthracene	C_14_H_10_	[M + H]	178.23	179.09	8.29	179.09, 171.12	Scrophulariae Radix
5.26	5-(Methylsulfanyl)penta nenitrile	C_6_H_11_NS	[M + H]	129.23	130.07	−10.67	130.07, 129.07, 126.95	Scrophulariae Radix
23.63	Sitogluside	C_35_H_60_O_6_	[M − H]	576.80	575.43	3.13	575.43, 461.29	Scrophulariae Radix
3.36	4-Methoxycinnamic acid	C_10_H_10_O_3_	[M + H]	178.18	179.07	3.71	146.06, 96.96	scrophulariae radix
16.08	Ningposide D	C_18_H_22_O_8_	[M + H]	366.40	367.14	1.00	275.20, 264.27	Scrophulariae Radix
25.32	5-(3-Hydroxypropyl)-2-methoxyphenol	C_10_H_14_O_3_	[M + H]	182.22	183.10	−1.66	167.08, 154.07	Scrophulariae Radix
0.60	Deltonin	C_45_H_72_O_17_	[M − H]	885.00	883.46	−1.23	268.80, 92.93	Scrophulariae Radix
4.49	5-(2-Hydroxyethyl)-2-methoxyphenol	C_9_H_12_O_3_	[M + H]	168.19	169.08	−2.76	169.09, 149.06, 132.08	Scrophulariae Radix
6.11	8-O-Feruloyl harpagide	C_25_H_32_O_13_	[M + H]	540.50	541.24	4.69	329.12, 126.03	Scrophulariae Radix

#### 3.5.3 Identification of the potential bioactive components of “Scrophulariae Radix–Fritillaria”

**FIGURE 8:**
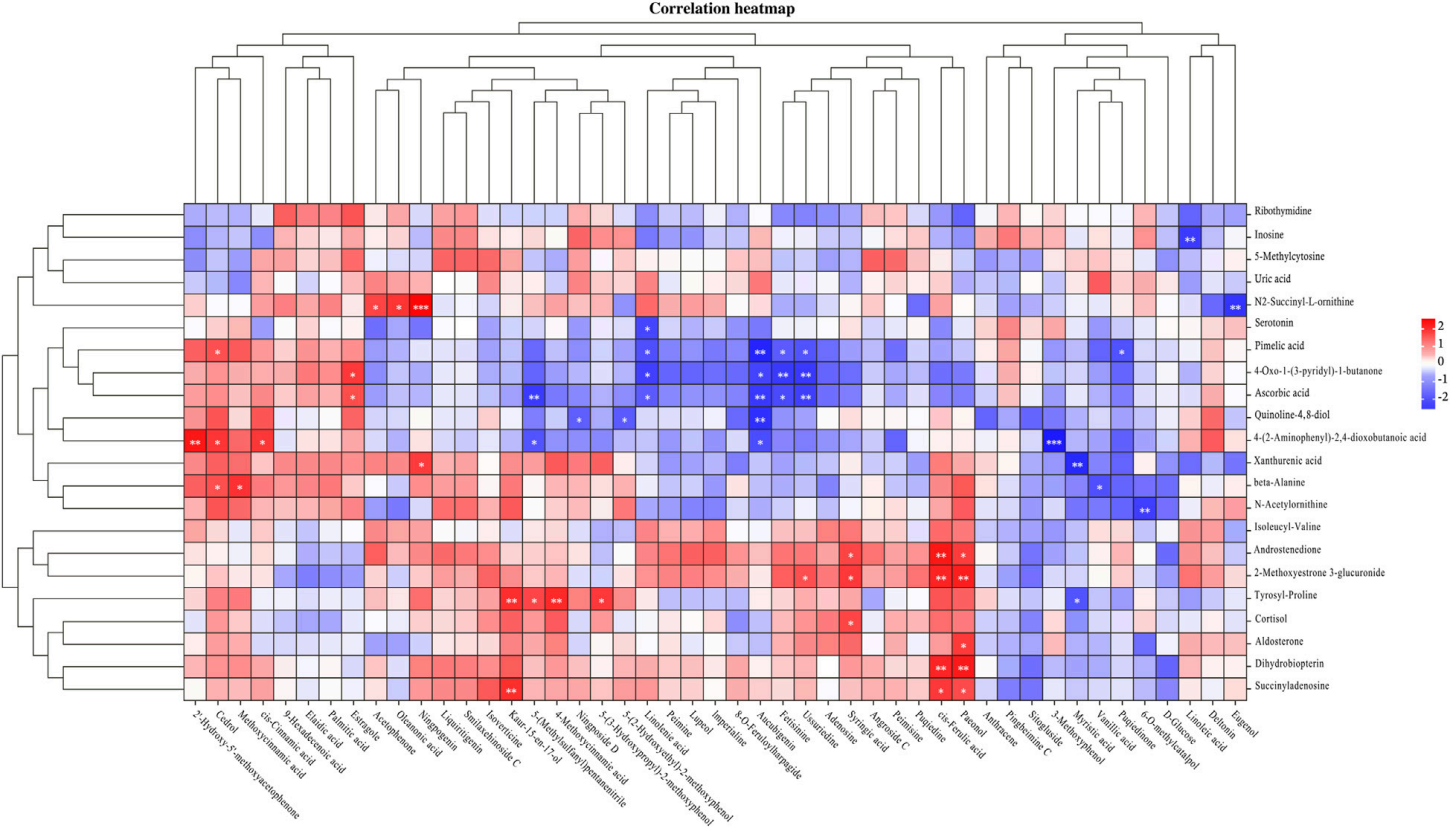
Heatmap of correlation analysis between the serum components of the “Scrophulariae Radix–Fritillaria” drug pair and urine biomarkers in the goiter rat model.

The correlation between the 22 urine biomarkers and 47 components of “Scrophulariae Radix–Fritillaria” identified in the serum of goiter model rats was evaluated by Pearson’s analysis (Figure 8). Nine components, namely, cedrol, cis-ferulic acid, fetisinine, linolenic acid, paeonol, ussuriedine, 5-(methylsulfanyl)pentanenitrile, aucubigenin, and syringic acid, were highly correlated with the urine biomarkers. Seven of these components were derived from Scrophulariae Radix and 2 from Fritillaria, which potentially mediate the therapeutic effects of the drug pair against goiter.

## 4 Discussion

Goiter has a high incidence and cancerization rate, and the number of cases is increasing year after year. Therefore, there is considerable interest in exploring its pathological basis in order to develop an effective therapeutic strategy. In this study, we analyzed the metabolic profile associated with goiter, and the changes induced by “Scrophulariae Radix–Fritillaria” in order to elucidate its mechanism of action. Clinically, 5-HT, NE, TSH, FT3, and FT4 are the diagnostic indicators of thyroid function. Aberrant changes in TSH levels disrupt the HPT, which in turn affects FT3 and FT4 levels and impairs thyroid function. Furthermore, FT3 negatively regulates the hypothalamic–pituitary–thyroid (HPT) axis through a feedback mechanism. Induction of goiter in a rat model decreased the serum levels of NE, FT3, and FT4 and increased those of 5-HT and TSH. Treatment with “Scrophulariae Radix–Fritillaria” restored these biochemical indices. We identified 22 biomarkers in the urine samples of goiter rats by metabolomics, of which 19 (uric acid, 4-oxo-1-(3-pyridyl)-1-butanone, inosine, xanthurenic acid, pimelic acid, ascorbic acid, succinyladenosine, ribothymidine, quinoline-4,8-diol, isoleucylvaline, 5-methylcytosine, serotonin, dihydrobiopterin, beta-alanine, tyrosyl-proline, androstenedione, cortisol, N2-succinyl-L-ornithine, and N-acetylornithine) were restored to normal levels by “Scrophulariae Radix–Fritillaria” and are mainly involved in the pathways of tryptophan metabolism, steroid hormone biosynthesis, and β-alanine metabolism.

Tryptophan metabolism: Tryptophan metabolism is involved in the pathogenesis of depression, enteritis, and other diseases (Nadeem et al., 2022; Wang et al., 2022). It is metabolized by tryptophan hydroxylase and aromatic amino acid decarboxylase in the 5-HT pathway. In the kynurenine pathway, tryptophan is metabolized by 2,3-dioxygenase and indoleamine 2,3-dioxygenase to kynurenine (Biswas et al., 2022; Zhen et al., 2022), which is eventually broken down into niacin, quinoline, and xanthurenic acid (Fukushima et al., 2022). In this study, the level of xanthurenic acid was lower and those of quinoline-4,8-diol and 4-(2-aminophenyl)-2,4-dioxobutanoic acid were higher in the urine of goiter rats compared to the controls, which may be related to abnormal kynurenine metabolism. Furthermore, the change in the serotonin content in goiter rats may have disrupted the 5-HT pathway, resulting in abnormal tryptophan levels. Treatment with “Scrophulariae Radix–Fritillaria” normalized the levels of these metabolites by targeting the tryptophan metabolism pathway.

Steroid hormone biosynthesis: Steroid hormone biosynthesis occurs in the adrenal cortex and gonads. In the adrenal cortex, cholesterol is used by hydroxysteroid dehydrogenase and cytochrome P450 to produce glucocorticoids and mineralocorticoids, respectively, which are involved in immune regulation, maintenance of internal homeostasis, and stress mitigation (Chang et al., 2021; Yang et al., 2021). Glucocorticoids formed at the end of the hypothalamic–pituitary–adrenal (HPA) axis include cortisol (Paschali et al., 2021; Kim et al., 2022). In addition, any changes in its levels can disrupt thyroid function by affecting the levels of FT3, FT4, and TSH (Doggui, 2016; Ernst et al., 2020). Fabrice et al. found that altered cortisol levels affect the function of the HPT axis (Duval et al., 2015). Aldosterone, a mineralocorticoid synthesized by aldosterone synthase from cholesterol, regulates the balance of electrolytes and water and is an important part of the renin–angiotensin–aldosterone system (RAAS) (Chandra et al., 2015). Thyroid dysfunction also affects the RAAS system and cortisol levels (Dizaye and Mustafa, 2019; Farhadi and Dizaye, 2019; Li et al., 2021). The thyroid and adrenal glands are regulated by the hypothalamic–pituitary axis to maintain normal metabolism, and any damage to the adrenal cortex can aggravate thyroid disease. In addition, the adrenal cortex also secretes sex hormones like dehydroepiandrosterone, which is the precursor of androstenedione. The latter is secreted by the gonads as well as the adrenal glands and is eventually converted to testosterone or estrone, which plays an important role in balancing androgens and estrogens (Agbaht and Gullu, 2014). Furthermore, androstenedione and 2-methoxyestrone 3-glucuronide also regulate the biosynthesis of steroid hormones in the gonads. According to our results, the decreased cortisol level in the urine of goiter rats may be related to the disruption of the HPA and HPT axes, leading to aberrant steroid hormone biosynthesis. In addition, decreased aldosterone content may interfere with the RAAS system, resulting in abnormal adrenal cortex function and steroid hormone biosynthesis.

Beta-alanine metabolism: β-alanine is the only naturally occurring β-type amino acid and is known to improve physical stamina (Matthews et al., 2019), enhance cognitive ability (Samadi et al., 2022), relieve fatigue (Horvath et al., 2016), and mitigate aging (Varanoske et al., 2021). It is mainly involved in the synthesis of pantothenic acid and coenzyme A. In addition, it is also the precursor of carnosine in the mammalian nervous system, which regulates the levels of thyroid hormones and has an inhibitory effect on thyroid cancer cells (Bao et al., 2015; Turner et al., 2021). The increased β-alanine content in the urine of goiter rats may lead to increased carnosine production, resulting in abnormal changes in thyroid hormones and thyroid function. “Scrophulariae Radix–Fritillaria” decreased the levels of β-alanine, suggesting that its therapeutic effects against goiter may be related to the β-alanine metabolic pathway. Liu et al. found that alanine metabolism is disturbed in patients with hypothyroidism (Liu et al., 2020). Likewise, Huang et al. reported aberrant alanine metabolism in the plasma of patients with thyroid nodules compared to the healthy controls (Huang et al., 2018), which is consistent with our study.

Through serum pharmacochemistry, we identified 38 prototype components of “Scrophulariae Radix–Fritillaria” in the serum of the treated animals, of which cedrol, cis-ferulic acid, fetisinine, linolenic acid, paeonol, ussuriedine, 5-(methylsulfanyl)pentanenitrile, aucubigenin, and syringic acid were correlated to urine metabolites and may therefore be potential bioactive compounds. Ling et al. found that the paeonol-platinum (II) complex promoted apoptosis of thyroid cancer cells, increased the fraction of sub-G1 cells, increased the expression of p27 and p21, downregulated p53 and cyclin D1, and inactivated the mTOR pathway in thyroid cancer cells (He et al., 2020). Panda et al. demonstrated that syringic acid, a thyroid hormone receptor-β agonist, protected rats against PTU-induced thyroid toxicity by increasing the levels of T4 and T3; decreasing TSH, TNF-α, IL-6, ALT and AST levels; and improving the histological characteristics of thyroid tissues (Panda et al., 2021). Most of the effective compounds of “Scrophulariae Radix–Fritillaria” are alkaloids, phenylpropanoids, iridoids, flavonoids, and other active ingredients (You-Hua et al., 2014), which may play a therapeutic role in thyroid diseases by inhibiting thyroid hormone synthesis, regulating LPAR3 level, and activating the PI3K–Akt pathway.

## 5 Conclusion

We were able to identify nine bioactive compounds of “Scrophulariae Radix–Fritillaria” in a rat model of goiter through UPLC-Q-TOF/MS-based metabolomics and serum pharmacochemistry. The therapeutic effects of the drug pair likely involve pathways related to tryptophan metabolism, steroid hormone biosynthesis, and beta-alanine metabolism. Our study provides an experimental basis for the clinical application of “Scrophulariae Radix–Fritillaria” in the treatment of goiter.

## Data Availability

The original contributions presented in the study are included in the article/Supplementary Material; further inquiries can be directed to the corresponding authors.
